# β-Escin overcomes trastuzumab resistance in HER2-positive breast cancer by targeting cancer stem-like features

**DOI:** 10.1186/s12935-022-02713-9

**Published:** 2022-09-20

**Authors:** Soeun Park, Jung Min Park, Minsu Park, Dongmi Ko, Seongjae Kim, Juyeon Seo, Kee Dal Nam, Eunsun Jung, Lee Farrand, Yoon-Jae Kim, Ji Young Kim, Jae Hong Seo

**Affiliations:** 1 Division of Medical Oncology, Department of Internal Medicine, Korea University College of Medicine, Korea University, Seoul, 02841 Republic of Korea; 2 Brain Korea 21 Program for Biomedical Science, Korea University College of Medicine, Korea University, Seoul, 02841 Republic of Korea; 3 Department of Biomedical Research Center, Korea University Guro Hospital, Korea University, 97 Gurodong-gil, Guro-gu, Seoul, 08308 Republic of Korea; 4 Adelaide Medical School, Faculty of Health and Medical Sciences, The University of Adelaide, Adelaide, South Australia 5000 Australia

**Keywords:** β-escin, Trastuzumab resistance, Drug repurposing, p95HER2, HER2-positive breast cancer, Cancer stem cells

## Abstract

**Background:**

The emergence of de novo or intrinsic trastuzumab resistance is exceedingly high in breast cancer that is HER2 positive and correlates with an abundant cancer stem cell (CSC)-like population. We sought to examine the capacity of β-escin, an anti-inflammatory drug, to address trastuzumab resistance in HER2-positive breast cancer cells.

**Methods:**

The effect of β-escin on trastuzumab-resistant and -sensitive cell lines in vitro was evaluated for apoptosis, expression of HER2 family members, and impact on CSC-like properties. An in vivo model of trastuzumab-resistant JIMT-1 was used to examine the efficacy and toxicity of β-escin.

**Results:**

β-escin induced mitochondrial-mediated apoptosis accompanied by reactive oxygen species (ROS) production and increased active p18Bax fragmentation, leading to caspase-3/-7 activation. Attenuation of CSC-related features by β-escin challenge was accompanied by marked reductions in CD44^high^/CD24^low^ stem-like cells and aldehyde dehydrogenase 1 (ALDH1) activity as well as hindrance of mammosphere formation. β-escin administration also significantly retarded tumor growth and angiogenesis in a trastuzumab-resistant JIMT-1 xenograft model via downregulation of CSC-associated markers and intracellular domain HER2. Importantly, β-escin selectively inhibited malignant cells and was less toxic to normal mammary cells, and no toxic effects were found in liver and kidney function in animals.

**Conclusions:**

Taken together, our findings highlight β-escin as a promising candidate for the treatment of trastuzumab-resistant HER2-positive breast cancers.

**Supplementary Information:**

The online version contains supplementary material available at 10.1186/s12935-022-02713-9.

## Introduction

Despite significant advances in treatment strategies and precision medicine, breast cancer remains a multifaceted and serious disease that accounts for approximately 15% of cancer mortality in women worldwide [1]. The HER2 oncogene is upregulated in 20–30% of breast cancers and is associated with more aggressive biological behavior, shorter disease-free intervals and poorer outcomes. The HER2-targeting antibody trastuzumab has shown significant clinical benefits in improving overall survival rates in patients with early-stage or metastatic breast cancer that is HER2-positive [2–4]. However, in HER2-overexpressing metastatic breast cancer patients, the emergence of de novo or intrinsic resistance to trastuzumab monotherapy is exceedingly high, ranging between 66 and 88% [5].

Trastuzumab resistance is a multifactorial phenomenon arising from the steric effects of p95HER2, heterodimerization of HER2 family members, activation of HER2 downstream signaling pathways, and the presence of cancer stem cells [6]. p95HER2 is a truncated form lacking the extracellular domain to which trastuzumab binds and elicits steric hindrance resulting in tyrosine kinase activity and survival signal transduction pathways. HER2 forms homo- or heterodimers with other HER family members including EGFR, HER3 and HER4, which initiate activation of the PI3K and MAPK pathways leading to cell survival, proliferation and avoidance of apoptosis [7–9]. In particular, HER2/HER3 dimers are highly mitogenic and significant mediators of survival signaling, and together they drive neoplastic cell transformation and mammary carcinoma growth [10, 11].

Cancer stem cells (CSCs) harbor the ability to self-renew and can differentiate into diverse lineages of cancer cells, a major cause of drug resistance, allowing cancer stem cells to survive and expand after chemotherapy [12, 13]. The capacity of CSCs to self-renew contributes to a survival advantage via the repair of DNA damage, while their differentiation properties promote tumorigenesis [13, 14]. The CD44^high^/CD24^low^ phenotype combined with high activity of aldehyde dehydrogenase 1 (ALDH1) are common hallmarks of breast cancer stem cells (BCSCs) [15, 16]. Considerable upregulation of the detoxifying enzyme ALDH1 confers aggressive properties and drug resistance to tumor cells after chemotherapy [17, 18]. The cell surface glycoprotein CD44 complexes with hyaluronan to mask its cognate epitope from binding to HER2, thereby facilitating trastuzumab resistance [6, 19]. In this context, a potential clinical strategy to address trastuzumab resistance is to simultaneously target cancer stem cells and tyrosine kinase activation of HER family members including the inactivation of steric effects.

β-escin is the primary active compound in horse chestnut (*Aesculus hippocastanum*) [20] and has anti-inflammatory and anti-edematous properties with an exceptional safety profile in clinical studies [21]. β-escin is known to exhibit anti-cancer effects in various cancer cell types via suppression of NF-kB activity, ROS production, induction of the intrinsic apoptotic pathway and G2/M arrest [22–24]. However, the antitumor efficacy and molecular mechanisms of β-escin on the HER2 signaling pathway and CSC-stem-like properties in HER2-positive breast cancer have not been elucidated. Herein, for the first time, we report the potent efficacy of β-escin, a drug repurposing candidate with an exceptional safety profile in addressing trastuzumab-resistant HER2-positive breast cancer.

## Materials and methods

### Reagents and antibodies

β-escin, propidium iodide (PI), Triton X-100 and dimethyl sulfoxide (DMSO) were manufactured by Sigma-Aldrich (St. Louis, MO). Primary antibodies were obtained for: Ki-67, CD31, ALDH1A1, CD44 and vimentin (Abcam, Cambridge, UK); HER2, HER3, phospho-HER2 (Y1221/1222), phospho-HER3 (Y1289), Akt, PARP, cleaved-caspase-3, Bcl-2, cleaved-PARP, cleaved-caspase-7 and Bax (Cell Signaling, Beverly, MA); cytochrome c and phospho-Akt (Santa Cruz Biotechnology, CA); anti-intracellular domain (ICD) HER2 clone 4B5 (Ventana Medical Systems, AZ); TOM 20 and GAPDH (Invitrogen, Carlsbad, CA). Secondary antibodies were HRP-conjugated anti-mouse and rabbit and IgG (Bio-Rad Laboratories, CA) and Alexa Fluor-594 and -488 goat anti-rabbit IgG (Invitrogen).

### Breast cancer cell lines

The human breast cancer cell lines SKBR3, BT474, MDA-MB-453 (ATCC; American Type Culture Collection), MDA-MD-231 (PerkinElmer, Inc. USA) and JIMT-1 (DSMZ GmbH, Germany) were cultured in DMEM, MEME or RPMI 1640 (Gibco, MD) containing 10% FBS and streptomycin-penicillin (100 U/mL). The human normal mammary epithelial MCF10A cell line (ATCC) was grown in MEGM supplemented with hEGF, hydrocortisone, insulin and bovine pituitary extract (SingleQuots™ Kit, Lonza, CA) with 100 U/mL streptomycin-penicillin. Cells were incubated at 37 °C in an atmosphere of 5% CO_2_. All human cell lines were validated by short tandem repeat profiling by Microgen Inc. (Seoul, South Korea).

### Generation of stabilized HER2- and high p95HER2-expressing MDA-MB-231 cells

HER2 and p95HER2 were stably overexpressed independently in MDA-MB-231 cells using a lentiviral vector, as described previously [25, 26].

### Cell viability assay

Cells were analyzed with a CellTiter 96® AQueous One Solution Cell Proliferation Assay according to the manufacturer’s protocol (Promega, Madison, WI). The concentration of formazan product was measured by at 490 nm using a microplate reader, 800TS (BioTek, VT) and analyzed using Gen5 software.

### Sub-G1 assessment and Annexin V assay

Cells were recovered and fixed using 95% ethanol with 0.5% Tween-20 for 24 h and incubated in propidium iodide (PI, 50 mg/mL) and 50 mg/mL RNase (30 min). The early and late apoptosis was analyzed with a FITC Annexin V Apoptosis Detection Kit (BD Biosciences), according to the manufacturer’s instructions. Stained cells were assessed using flow cytometry with a BD LSRFortessa™ X-20 Cell Analyzer (BD Biosciences).

### CD44/CD24 staining and Aldefluor-positivity assay

ALDH1 activity was analyzed with an Aldefluor assay kit (Stem Cell Technologies, Canada) according to the manufacturer’s instructions. For 45 min (37 ℃), cells were incubated in Aldefluor assay buffer containing ALDH protein substrate BODIPY-aminoacetaldehyde (BAAA, 1 µM/0.5 × 10^6^ cells). The ALDH1-specific inhibitor diethylamino-benzaldehyde (DEAB; 50 mM) was defined as the baseline of Aldefluor fluorescence with flow cytometry using a BD Cell Analyzer. For CD44/CD24 analysis, cells (1﻿ × 10^6^) were immunostained with FITC-conjugated anti-CD24 or PE-conjugated anti-mouse IgG and PE-conjugated anti-CD44 antibodies (BD Biosciences) at 4 °C (30 min) and analyzed by flow cytometry.

### Western blot

Cells were lysed in cold lysis buffer [0.5% Triton X-100, 30 mM NaCl, 50 mM Tris–HCl (pH 7.4)] containing protease and phosphatase inhibitor cocktail tablets. Cell supernatant was recovered after centrifugation (14,000*g* at 4 °C for 20 min) and protein concentrations were quantified with a Quick Start™ Bradford Protein Assay (Bio-Rad). Equal amounts of protein (25 μg) were separated by SDS-PAGE and transferred to PVDF membranes (Millipore, Burlington, MA). After blocking with 5% skim milk for 30 min, membranes were incubated overnight at 4 °C with primary antibodies diluted with 5% BSA [HER2 (1:2000), phospho-HER2 (1:1000), HER3 (1:2000), phospho-HER3 (1:2000), Akt (1:2000), phospho-Akt (1:2000), Bcl-2 (1:2000), Bax (1:2000), cleaved-PARP (1:2000), PARP (1:2000), cleaved-caspase-7 (1:2000) cleaved-caspase-3 (1:2000), or GAPDH (1:3000)], followed by incubation with HRP-conjugated mouse or rabbit secondary antibodies (1:3000–1:10,000). Protein signal intensity was measured with an ECL Western Blotting Substrate (Thermo Fisher) on X-ray film (Agfa Healthcare, Belgium) and quantitated with AlphaEaseFC software (Alpha Innotech, CA).

### Reactive oxygen species generation

Cells were treated with β-escin (20 or 30 μM) for 3 and 6 h. The cells were then stained with 20 μM 2′, 7′-Dichlorodihydrofluorescin diacetate (DCFH-DA; Cell Biolabs, San Diego, CA) for 30 min at 37 °C. Fluorescence 2′, 7′- Dichlorodihydrofluorescein (DCF) oxidized by intracellular ROS was measured at 480 nm excitation and 530 nm emission using a BD LSRFortessa™ X-20 Cell Analyzer using FlowJo software.

### Immunocytochemistry

Cells were fixed in 8-well chamber slides (BD Biosciences) with 4% paraformaldehyde, washed with PBS, and incubated with 0.2% Triton X-100 (10 min). The cells were then incubated with primary antibodies [HER2 (1:100), cytochrome c (1:100) and TOM 20 (1:100)] in antibody diluent (Dako, Santa Clara, CA) overnight at 4 ℃, before the addition of an Alexa Fluor^®^-594 conjugated secondary antibody. The cells were mounted using Antifade Mount plus DAPI (Invitrogen) and images were acquired with a confocal microscope (Carl Zeiss, Germany).

### In vitro* mammosphere formation assay*

JIMT-1 (1.5 × 10^4^/mL) and BT474 (5 × 10^4^/mL) cells were plated using ultralow attachment dishes and grown in HuMEC basal serum free medium (Gibco) with B27 (1:50, Invitrogen), 20 ng/mL human epidermal growth factor (EGF, Sigma-Aldrich), 20 ng/mL basic fibroblast growth factor (bFGF, Sigma-Aldrich), 1% antibiotic–antimycotic, 4 μg/mL heparin, and 15 μg/mL gentamycin at 37 °C (5% CO_2_). The number and volumes of the mammospheres was assessed with a CKX53 inverted microscope (Olympus Life Science). Mammosphere volumes were calculated by the formula volume = 4/3*3.14(π)*r^3^ (r: radius).

### Xenograft experiments

All animal procedures were conducted according to the *Guide for the Care and Use of Laboratory Animals*, with the approval of Korea University Institutional Animal Care and Use Committee (IACUC, KOREA-2021–0070). Female BALB/c nude mice (5 weeks old) were purchased from NARA Biotech (Seoul, Korea) and kept in a specific pathogen-free environment. JIMT-1 cells (4 × 10^6^) were inserted into the fourth mammary fat pads of the BALB/c nude female mice after one-week acclimation (n = 8/each group). When average tumor volumes reached 100 mm^3^, test animals were randomized into 2 groups (n = 8/each group), with solvent control (DMSO/PBS, 1:9) or β-escin (4 mg/kg, 5 times per week) and administered intraperitoneally for 28 days. Tumor volumes and body weight were measured twice per week after the first treatment, and tumor volumes were calculated with the formula V = (Length × Width^2^)/2.

### Serum biochemistry for liver and renal injury biomarkers

After sacrifice, blood samples were collected from each animal, and serum activities of alanine aminotransferase (ALT), aspartate aminotransferase (AST) and blood urea nitrogen (BUN) levels were assessed with an assay kit following the manufacturer’s protocol (Sigma-Aldrich). All assays were measured with a Spectra Max 190 (Molecular devices, CA) and analyzed using SoftMax Pro 7 software.

### Immunohistochemistry with apoptosis in-situ localization (TUNEL)

After removal, tumors were fixed in 10% neutral-buffered formalin before paraffin embedding. Tissue Sects. (5 µm) were then placed on positively-charged glass slides before deparaffinization with xylene and dehydrated via a graded alcohol series to water. Sections were boiled in citric acid buffer for antigen retrieval (pH 6.0). The tissue sections with primary antibodies [Ki-67 (1:100), HER2 (1:100), CD31 (1:100), 4B5 (1:100), CD44 (1:100), ALDH1A1 (1:100) and vimentin (1:300)] in antibody diluent were incubated overnight (4 °C) before reaction with Alexa Fluor-conjugated secondary antibodies (Alexa Fluor® 488- or 594, RT; 2 h) followed by Antifade Mount plus DAPI. TUNEL assay was performed with tissue sections using an In Situ Cell Death Detection TUNEL kit (Roche, Switzerland) in accordance with the manufacturer’s protocol.

### Wound healing assessment

To analyze kinetic migration, JIMT-1 cells were grown to ~ 90% confluency in 96-well plates (Essen ImageLock, MI). Physical wounds were created using a 96-pin Wound Maker device before washing in media to prevent reattachment of removed cells. Cells were treated with β-escin after wound creation. The wound fields were monitored, and an image captured each hour up to 24 h using an IncuCyte™ ZOOM® live-cell Imaging System (Essen BioScience). Wound confluency was assessed by the IncuCyte™ Scratch Wound Analysis Software Module.

### Statistical analysis

Data was analysed with GraphPad Prism 9.0 (San Diego, CA). Results are shown as mean ± SEM after at least three independent experiments. Data was assessed by student’s *t*-test, and ANOVA (one- or two-way) as appropriate. Significance between multiple groups was determined with the Bonferroni’s *post-hoc* test, defined at *p* < 0.05.

## Results

### β-escin reduces cell viability and increases apoptosis in HER2-positive breast cancer cells

**Fig. 1:**
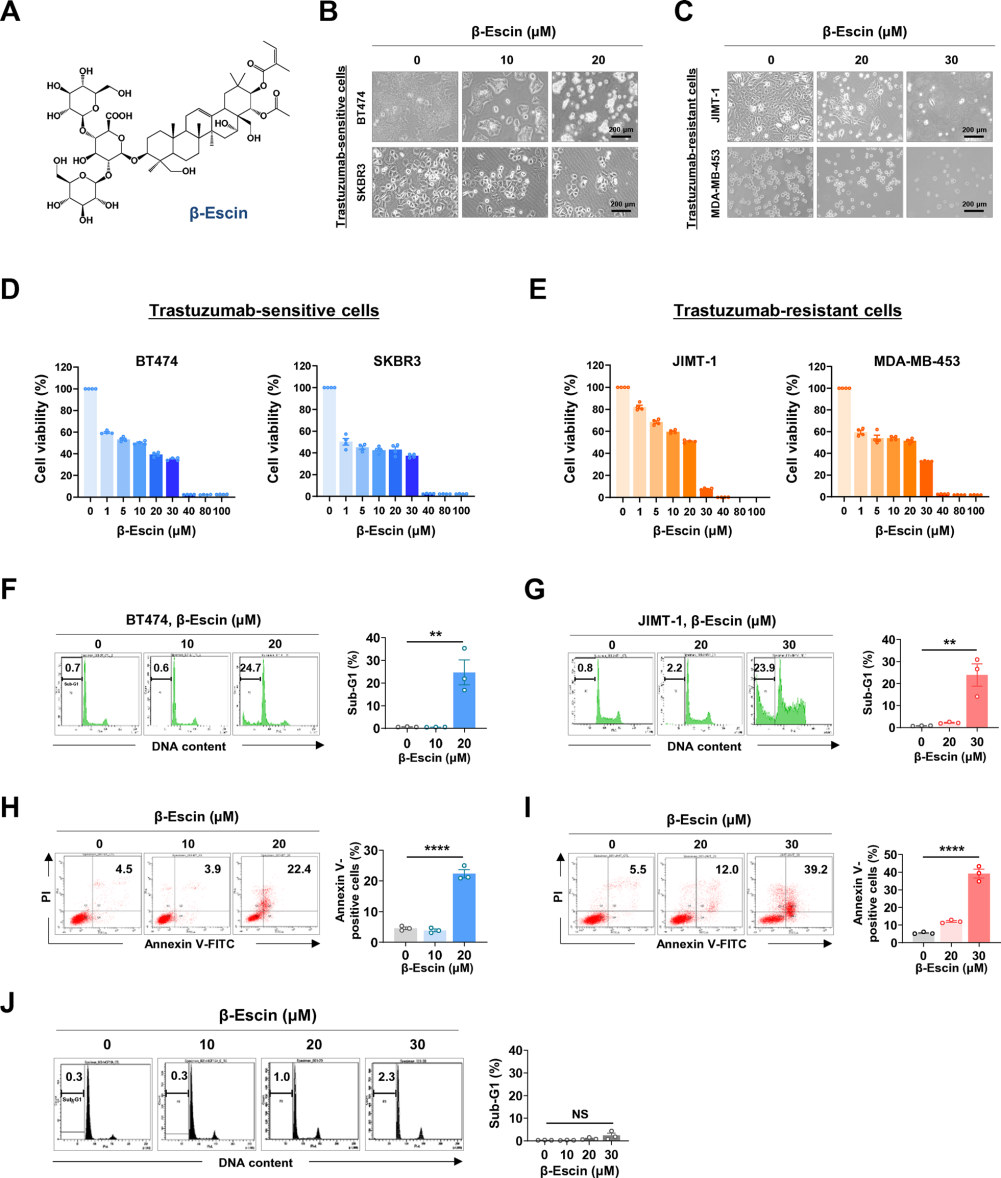
β-escin induces apoptosis in trastuzumab-sensitive and –resistant cells. **A** Chemical structure of β-escin. **B** The changes in morphology of BT474 and SKBR3 cells after treatment of β-escin (10-20 μM, 48 h) as observed by phase-contrast microscopy. **C** Representative phase-contrast images of JIMT-1 and MDA-MB-453 cells after treatment with β-escin (10-30 μM, 48 h). **D**, **E** Trastuzumab-sensitive SKBR3 and BT474 cells (**D**) and trastuzumab-resistant MDA-MB-453 and JIMT-1 cells (**E**) were treated with various concentrations of β-escin (1-100 μM) for 48 h, and cell viability was evaluated by MTS assay (****p<0.0001). **F**, **G** BT474 (**F**) and JIMT-1 cells (**G**) were treated with β-escin (10-20 μM and 20-30 μM, respectively) for 48 h, and the percentages of cells in the sub-G1 phase were quantified using flow cytometry (**p<0.01). **H**, **I** The percentages of the early and late apoptotic cells in BT474 (**H**) and JIMT-1 cells (**I**) following exposure to β-escin (10-20 μM and 20-30 μM, respectively) were determined by annexin V/PI staining (****p<0.0001). **J** Normal human mammary gland epithelial MCF10A cells were treated with β-escin (10-30 μM) for 48 h, and the sub-G1 fraction was analyzed (not significant, NS). The results are expressed as mean ± SEM after three independent experiments and analyzed by one-way ANOVA and Bonferroni’s post hoc test

Escin is a pentacyclic triterpene saponins and exists as α and β isomers. β-escin is the primary active compound that consists of a hydrophobic aglyconic part (pentacyclic triterpene) harboring corticosteroid-like activity and a hydrophilic glyconic part with glucuronic acid and glucose (Fig. 1A). We first sought to investigate the effect of β-escin on cell viability and apoptosis in HER2-positive breast cancer cells, trastuzumab-sensitive BT474 and SKBR3 cells, and trastuzumab-resistant JIMT-1 and MDA-MB-453 cells. The cells were treated with β-escin or DMSO (control vehicle) at varying concentrations for 48 h. β-escin (1-100 μM, 48 h) significantly reduced cell viability in a dose-dependent manner in both trastuzumab-sensitive and -resistant cells (****p<0.0001, Fig. 1D and E). From concentrations of 40 μM, cells exhibited severe toxicity, thus we chose an optimal concentration range of between 10 and 30 μM for further experiments investigating apoptosis, immunoblot analysis, and cancer stem-like properties. The cells treated with β-escin (10-30 µM, 48 h) showed typical morphological features of cytotoxicity with cytosolic shrinkage (Fig. 1B and C). Flow cytometry analyses revealed that β-escin induced a marked accumulation in the sub-G1 population and a significant increase in the number of early and late apoptotic cells in trastuzumab-sensitive BT474 (**p<0.01 and ****p<0.0001, Fig. 1F and H, respectively) and -resistant JIMT-1 cells (**p<0.01 and ****p<0.0001, Fig. 1G and I, respectively). The cell population of the sub-G1 phase in human normal mammary epithelial MCF10A cells had no statistically significant alterations at concentrations up to 30 μM (NS, Fig. 1J). Our data implies that β-escin selectively causes apoptotic cell death in tumor cells and was not toxic in normal cells.

### β-escin-induced apoptosis is concomitant with caspase-3/-7 activation and mitochondrial events

**Fig. 2:**
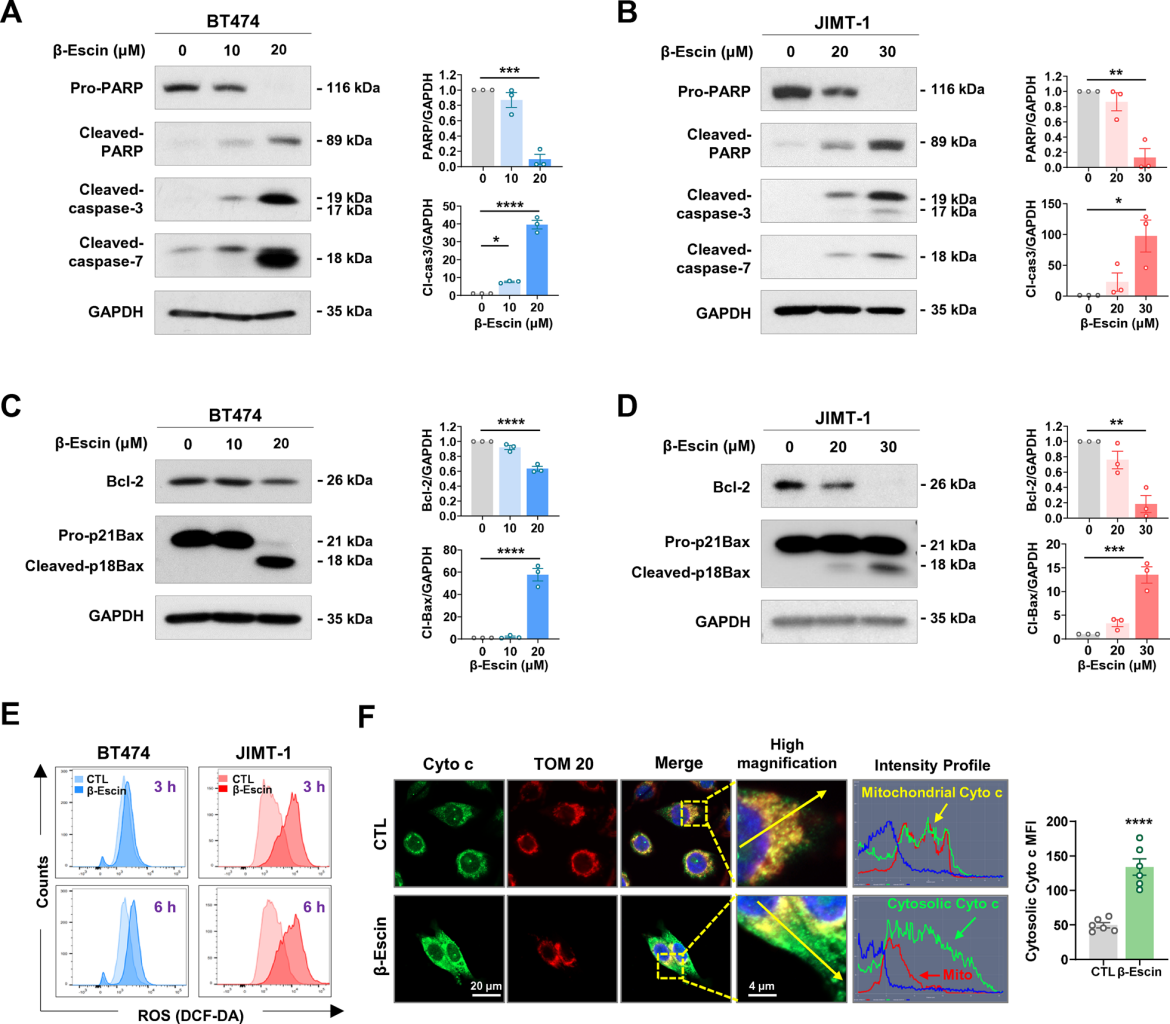
β-escin-induced apoptosis is associated with activation of caspases and dysregulation of mitochondrial proteins. **A**, **B** The effects of β-escin (10-30 μM, 48 h) on apoptotic-related proteins. BT474 and JIMT-1 cells were treated with β-escin (10-20 μM and 20-30 μM, respectively) for 48 h. The expression of cleaved-caspase-7, cleaved-caspase-3, and cleaved-PARP were upregulated in both BT474 (**A**) and JIMT-1 cells (**B**). Quantitative graphs of protein content are shown in the right panels (*p<0.05). **C**, **D** The effects of β-escin (10-30 μM, 48 h) on the expression of Bcl-2 family proteins including Bcl-2 and Bax in BT474 (**C**) and JIMT-1 cells (**D**). Quantitative graphs represent the ratio of Bcl-2/GAPDH and cleaved-Bax/GAPDH (right panels, **p<0.01). Results are shown as mean ± SEM of at least three independent experiments. Data were analyzed by one-way ANOVA and Bonferroni’s post hoc test. **E** β-escin increases ROS accumulation. BT474 and JIMT-1 cells were treated with β-escin (20 or 30 µM, respectively) for 3 and 6 h. The cells were stained with DCF-DA and analyzed by flow cytometry. **F** β-escin induces release of cytochrome c. JIMT-1 cells were treated with β-escin at 30 µM for 24 h and immunostained for cytochrome c (green) and TOM 20 (red) with DAPI (nuclei, blue). The cytochrome c intensity (green line) and cellular localization (yellow or green arrows) were analyzed with confocal microscopy using the intensity profile tool (straight yellow line). The mean fluorescence intensity (MFI) representing cytosolic cytochrome c were measured using the histogram tool (****p<0.0001, right panel). Cyto c, cytochrome c; TOM 20, translocase of outer mitochondrial membrane 20

It is known that β-escin induces apoptosis via mitochondrial dysfunction such as loss of membrane potential and the induction of oxidative stress [27]. To investigate whether β-escin induces the intrinsic apoptosis pathway in HER2-positive breast cancer cells, we examined mitochondrial events, including activation of effector caspases, expression of pro- and anti-apoptotic mitochondrial proteins, ROS production and cytochrome c release. Exposure to β-escin (10-30 μM, 48 h) resulted in the activation of caspase-3 and caspase-7 leading to subsequent cleavage of PARP in BT474 (*p<0.05, Fig. 2A) and JIMT-1 cells (*p<0.05, Fig. 2B). β-escin-induced apoptosis was accompanied by increased ROS production at an early stage (3-6 h) (Fig. 2E). Deregulation of mitochondrial proteins was observed in the presence of β-escin, as evidenced by Bcl-2 downregulation and increased cleaved Bax fragments at 18 kDa, representing the active form (**p<0.01, Fig. 2C and D). To determine whether these mitochondrial alterations induced cytochrome c release, we further performed double-immunocytochemistry for cytochrome c and the translocase of outer membrane 20 (TOM 20) as a mitochondrial marker. The green signal intensity profile represents the subcellular localization of cytochrome c with remarkable release from the mitochondria into the cytoplasm in the presence of β-escin (30 μM, 24 h, Fig. 2F). Furthermore, quantification of the mean fluorescence intensity (MFI) suggests that cytosolic cytochrome c was significantly increased after β-escin challenge (right panel, ****p<0.0001). Our findings suggest that the β-escin-induced intrinsic apoptosis pathway is initiated by excessive ROS generation at an early stage as well as the induction of disproportionate Bcl-2 and Bax levels, thus resulting in the release of cytochrome c and effector caspase-3/-7 activation.

### β-escin downregulates HER2/HER3/Akt and truncated p95HER2

**Fig. 3:**
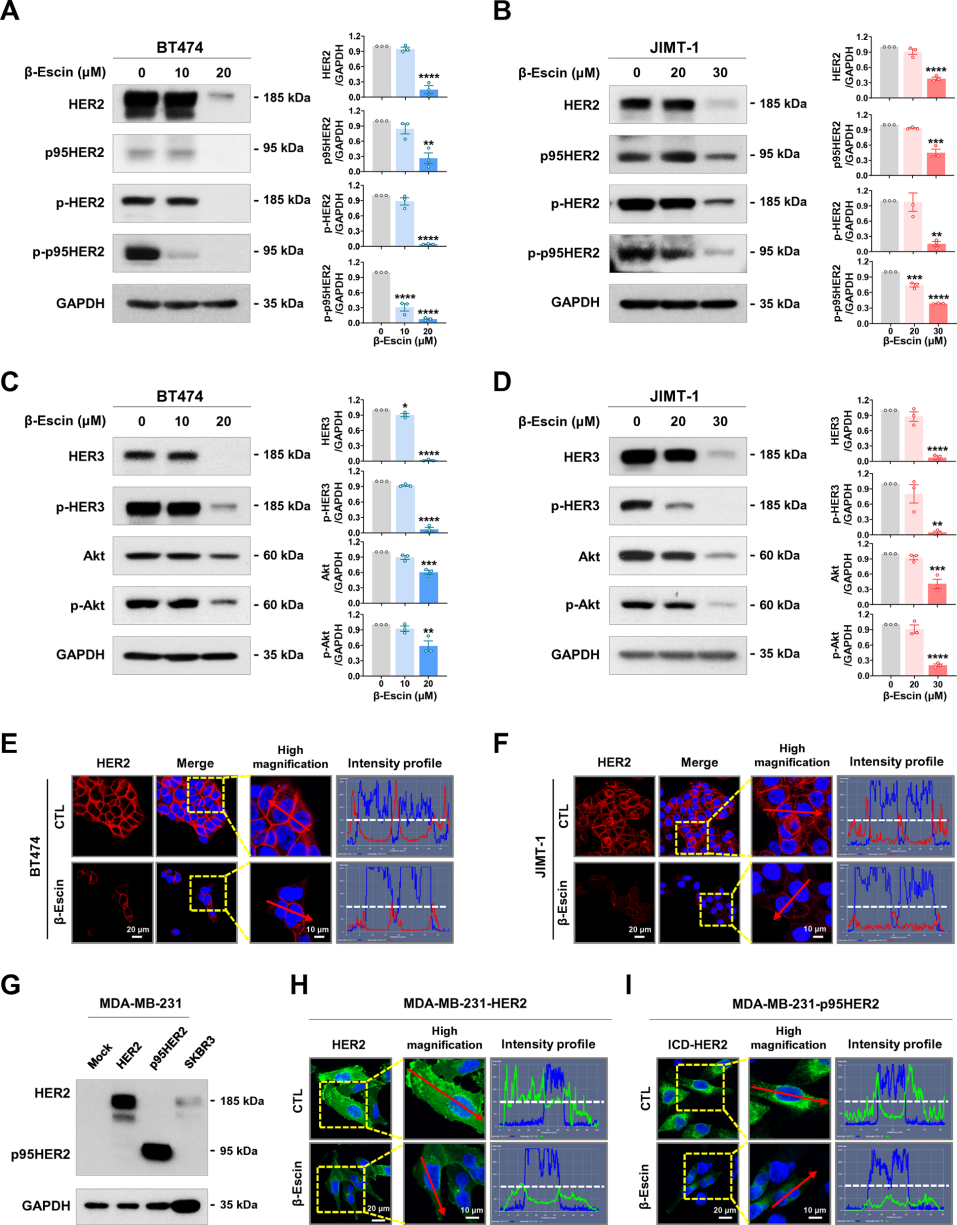
β-escin downregulates HER2, p95HER2, HER3 and Akt expression. **A**–**D** Western blots for HER2, p95HER2, phospho-HER2 (Y1221/1222), phospho-p95HER2, HER3, phospho-HER3 (Y1289), Akt, and phospho-Akt expression in BT474 (**A**, **C**) and JIMT-1 cells (**B**, **D**) following exposure to β-escin (10-20 μM and 20-30 μM, respectively) for 48 h. GAPDH was used as an internal loading control. The ratio of the protein content is represented in the right panels (*p<0.05). (**E**, **F**) Immunofluorescence analysis of HER2 (red) with DAPI (nuclei, blue) in BT474 (**E**) and JIMT-1 cells (**F**) after treatment with β-escin (20 and 30 µM, respectively) for 24 h. Intensity profiles represent HER2 expression with green signal fluorescence. **G** Immunoblot analyses of HER2 and p95HER2 protein content in HER2- and p95HER2-overexpressing MDA-MB-231, and SKBR3 cells. (**H**, **I)** Immunofluorescence analyses of HER2 (green) and ICD-HER2 (green, 4B5) with DAPI (nucleus, blue) in HER2- and p95HER2-overexpressing MDA-MB-231 cells in the presence of β-escin (20 and 30 µM, respectively) for 24 h. Intensity profiles were analyzed with the histogram tool in the Zen blue software and the horizontal line (white dotted line) indicates 100 intensity units (range of y-axis, 0-250 units)

We next evaluated whether β-escin affects expression of HER2 family members in HER2-positive breast cancer cells. Treatment with β-escin (10-30 μM, 48 h) significantly decreased total and phosphorylated HER2 (Y1221/1222) and HER3 (Y1289) with subsequent downregulation of Akt and phospho-Akt in both trastuzumab-sensitive BT474 (*p<0.05, Fig. 3A and C) and -resistant JIMT-1 cells (**p<0.01, Fig. 3B and D). β-escin treatment also caused downregulation of truncated p95HER2 and phospho-p95HER2 (Fig. 3A and B) which elicits the tyrosine kinase activity thought to be responsible for trastuzumab resistance. Immunocytochemical analysis further supported the notion that β-escin (30 μM, 24 h) induces a remarkable reduction in HER2 expression in both trastuzumab-sensitive BT474 (Fig. 3E and Additional file 1: Fig. S1A) and -resistant JIMT-1 cells (Fig. 3F and Additional file 1: Fig. S1B).

To confirm that β-escin downregulates HER2 and p95HER2, full-length HER2- and p95HER2-overexpressing MDA-MB-231 cells (Fig. 3G) were treated with β-escin (30 μM, 24 h). Intensity profiling revealed that HER2 or ICD-HER2 was highly expressed in the plasma membrane with green fluorescent signal in the MDA-MB-231-HER2 and MDA-MB-231-p95HER2 cells, respectively, while their expression was markedly diminished in the presence of β-escin (Fig. 3H, I and Additional file 1: Fig. S1C, D).

### β-escin hampers BCSC-like properties and migratory ability

**Fig. 4:**
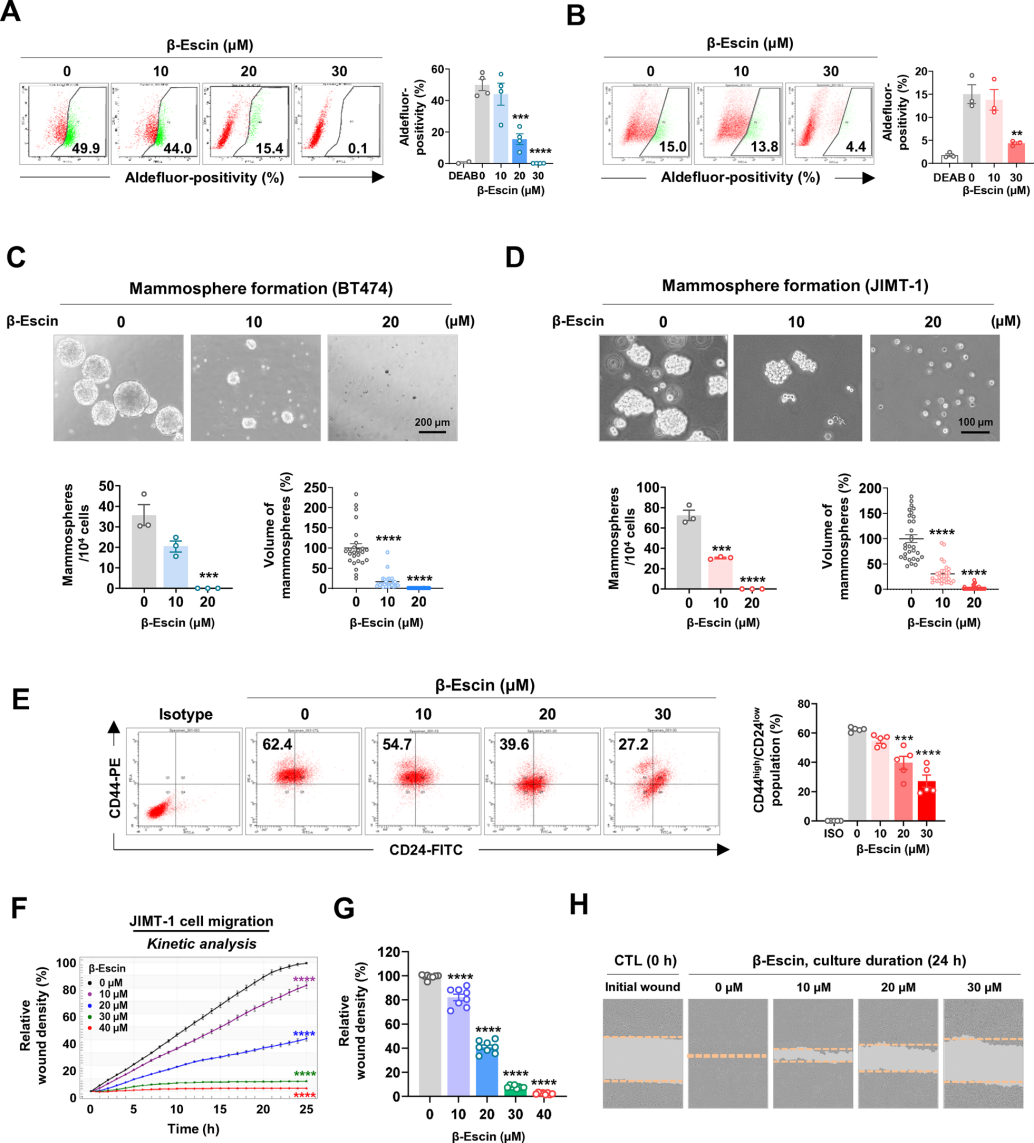
β-escin impairs CSC-like properties. **A**, **B** BT474 and JIMT-1 cells were treated with β-escin (10-30 μM) for 48 h and ALDH1 activity was assessed by flow cytometry. DEAB was used to define the baseline of Aldefluor-positive fluorescence. The quantitative graphs represent the percentage of Aldefluor-positive cells in BT474 (A, ***p<0.001) and JIMT-1 cells (B, **p<0.01). **C**, **D** BT474 (C, 5×10^4^ cells/ml) and JIMT-1 (D, 1.5×10^4^ cells/ml) were treated with β-escin (10-20 μM) in serum-free suspension conditions for 4 and 8 days, respectively. The number and volume of mammospheres were significantly reduced in the presence of β-escin, and the quantitative graphs are shown in the bottom panels, respectively (***p<0.001). **E** Effect of β-escin (10-30 μM, 48 h) on the CD44^high^/CD24^low^ stem-like phenotype in JIMT-1 cells. CD44^high^/CD24^low^ populations were determined by flow cytometry and were quantified (right panel, ***p<0.001). **F** JIMT-1 cells were treated with β-escin (0-40 μM) for 24 h. Cell migration was kinetically monitored with an IncuCyte™ System and quantified for the indicated time durations (****p<0.0001). **G** The quantitative graph represents the percentages of relative wound density of JIMT-1 cells at 24 h (****p<0.0001). **H** Representative images of wound closure by cell migration at 0 and 24 h after β-escin treatment (0-30 μM). The orange dotted line indicates the edge of the scratched wound

Cumulative evidence suggests that the occurrence of cancer stem cells in HER2-positive breast cancers is linked with tumor aggression and trastuzumab resistance [28, 29]. To explore the influence of β-escin on cancer stem-like properties in HER2-positive breast cancer cells, ALDH1 activity and the CD44^high^/CD24^low^ phenotype were analyzed, as well as *in vitro* mammosphere-forming ability. A CSC biomarker, ALDH1 is also a putative marker of resistance to chemotherapeutic drugs and promotes tumor stemness and cancer progression [15, 30]. Aldefluor-positivity assessment revealed that ALDH1 activity was dose-dependently reduced after exposure to β-escin (10-30 μM, 48 h) in both BT474 (***p<0.001, Fig. 4A) and JIMT-1 (**p<0.01, Fig. 4B). Mammospheres harbor plentiful mammary stem-like cells and the sphere formation assay is an efficient tool for evaluating the effect of CSC targeting drugs [31]. We have previously reported that stemness-related proteins including ALDH1, Nanog, Oct4, and Sox2 were higher in mammospheres compared to their adherent counterparts [32]. Formation of mammospheres derived from BT474 and JIMT-1 cells was markedly attenuated in response to β-escin treatment (10-20 μM), as evidenced by a significant decrease in the number and volume of mammosphere formed under anchorage-independent serum-free culture conditions (***p<0.001, Fig. 4C and D).

The mesenchymal-like CD44^high^/CD24^low^ population is an aggressive phenotype, that is highly tumorigenic and more resistant to drugs [28]. The CD44^high^/CD24^low^ phenotypes account for approximately 0.5% of trastuzumab-sensitive BT474 cells, whereas they account for more than 50% in trastuzumab-resistant JIMT-1 cells [32, 33]. In the present study, the CD44^high^/CD24^low^ subpopulations of JIMT-1 cells were as high as 63.4%, and these cell populations were significantly reduced by β-escin challenge (***p<0.001, Fig. 4E). CD44 is a key regulator in the rearrangement of membrane-associated cytoskeletal components, and participates in cell adhesion, integrate, motility and invasiveness [34, 35]. We next explored whether β-escin affects the migratory ability of trastuzumab-resistant HER2-positive breast cancer cells. A kinetic analysis of cell migration was performed in JIMT-1 cells following β-escin treatment (0-40 µM, 24 h). β-escin significantly reduced the migration of JIMT-1 cells in a dose-dependent manner (****p<0.0001, Fig. 4F–H), suggesting that it could contribute to preventing cell dissemination of trastuzumab-resistant cells with high CD44 expression.

### β-escin retards tumor growth in trastuzumab-resistant JIMT-1 xenografts

**Fig. 5:**
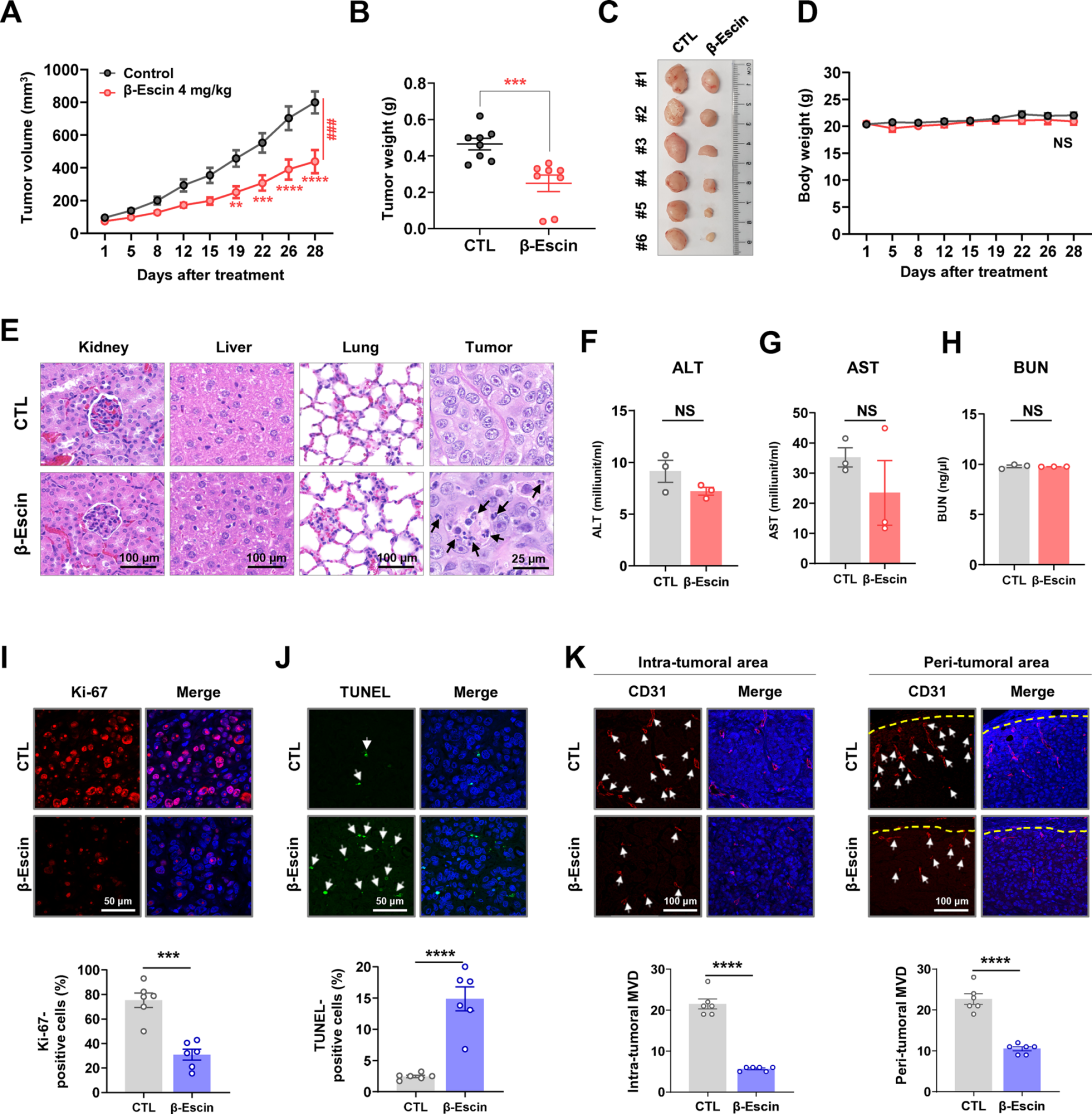
β-escin suppresses growth of JIMT-1-derived xenografts. **A**–**D** Impact of β-escin on tumor growth. JIMT-1 cells (4×10^6^) were injected into the fourth mammary fat pad of BALB/c nude mice. Animals were intraperitoneally administered with β-escin (4 mg/kg, body weight, 5 times/week for 4 weeks). β-escin administration resulted in a significant decrease in tumor volume (**A**, **p<0.01, n = 8) and tumor weight (**B**, ***p<0.001, n = 8). **C** The tumor burden was markedly reduced in the β-escin-treated group. **D** No significant alteration in body weight between the β-escin and CTL groups was observed (NS; not significant, n = 8). (**E**) Representative images of H&E staining in kidney, liver, lung and tumor tissues from the β-escin and CTL groups. The black arrows indicate the apoptotic cells with cell shrinkage and nuclear condensation. **F**–**H** Blood biochemical analyses for hepatic and renal function revealed no significant changes in ALT (**F**), AST (**G**), and BUN (**H**) by β-escin administration. ALT, alanine aminotransferase; NS, not significant; AST, aspartate aminotransferase; BUN, blood urea nitrogen. **I** The effect of β-escin on Ki-67 expression. Sections were immunostained to assess Ki-67 (red) and DAPI (blue) and the percentages of Ki-67-positive cells were quantified (bottom panel, ***p<0.001). **J** β-escin-induced apoptosis was determined by TUNEL assay. The white arrows indicate the TUNEL-positive apoptotic cells, and the percentages of TUNEL-positive cells were quantified (****p<0.0001). **K** Tumor angiogenesis was evaluated for each group of xenograft tumors. Tissues were immunostained using an endothelial cell marker CD31 (red) and DAPI (blue). The number of CD31-positive microvessels in the intra-tumoral (****p<0.0001) and peri-tumoral areas (****p<0.0001) were quantified, respectively

JIMT-1 cells (4 x 10^6^) were injected into the fourth mammary gland fat pads of 6-week-old female BALB/c nude mice, and β-escin (4 mg/kg·BW) or vehicle control (DMSO/PBS, 1:9) was administered 5 times per week for 4 weeks after the tumor volume reached approximately 100 mm^3^ in size. β-escin administration caused significant inhibition of tumor growth (**p<0.01, Fig. 5A, n=8, each group), tumor weight (***p<0.001, Fig. 5B) and tumor burden (Fig. 5C). There was no significant difference in body weight between the β-escin-treated and control groups (NS, Fig. 5D). Representative histological images for H&E staining revealed that the tumor tissue of the β-escin-treated group exhibited greater cell shrinkage and nuclear condensation (black arrow) compared with their control counterparts, while there were no histologically significant alterations in the kidney, liver and lung (Fig. 5E). We further employed blood biochemical assays for ALT, AST and BUN to evaluate the potential organ toxic effects of β-escin. There were no statistically significant changes in ALT, AST, or BUN levels in serum (NS, Fig. 5F–H), suggesting that β-escin had no observable effects on hepatic and renal functions.

The inhibitory effects of β-escin on tumor growth were accompanied by a decrease in Ki-67 index (***p<0.001, Fig. 5I and Additional file 1: Fig. S2A) and a significant increase in apoptosis as determined by TUNEL-positivity representing DNA fragments (****p<0.0001, Fig. 5J and Additional file 1: Fig. S2B). To further assess the effect of β-escin on angiogenesis, we performed a microvessel density assay with a specific endothelial adhesion molecule CD31. The number of CD31-positive microvessels in both intra-tumoral and peri-tumoral areas was markedly reduced in the β-escin-treated groups compared with their control counterparts (****p<0.0001, Fig. 5K and Additional file 1: Fig. S2C).

### β-escin downregulates HER2, ICD-HER2, and BCSC-related markers in JIMT-1-derived xenograft tumors

**Fig. 6:**
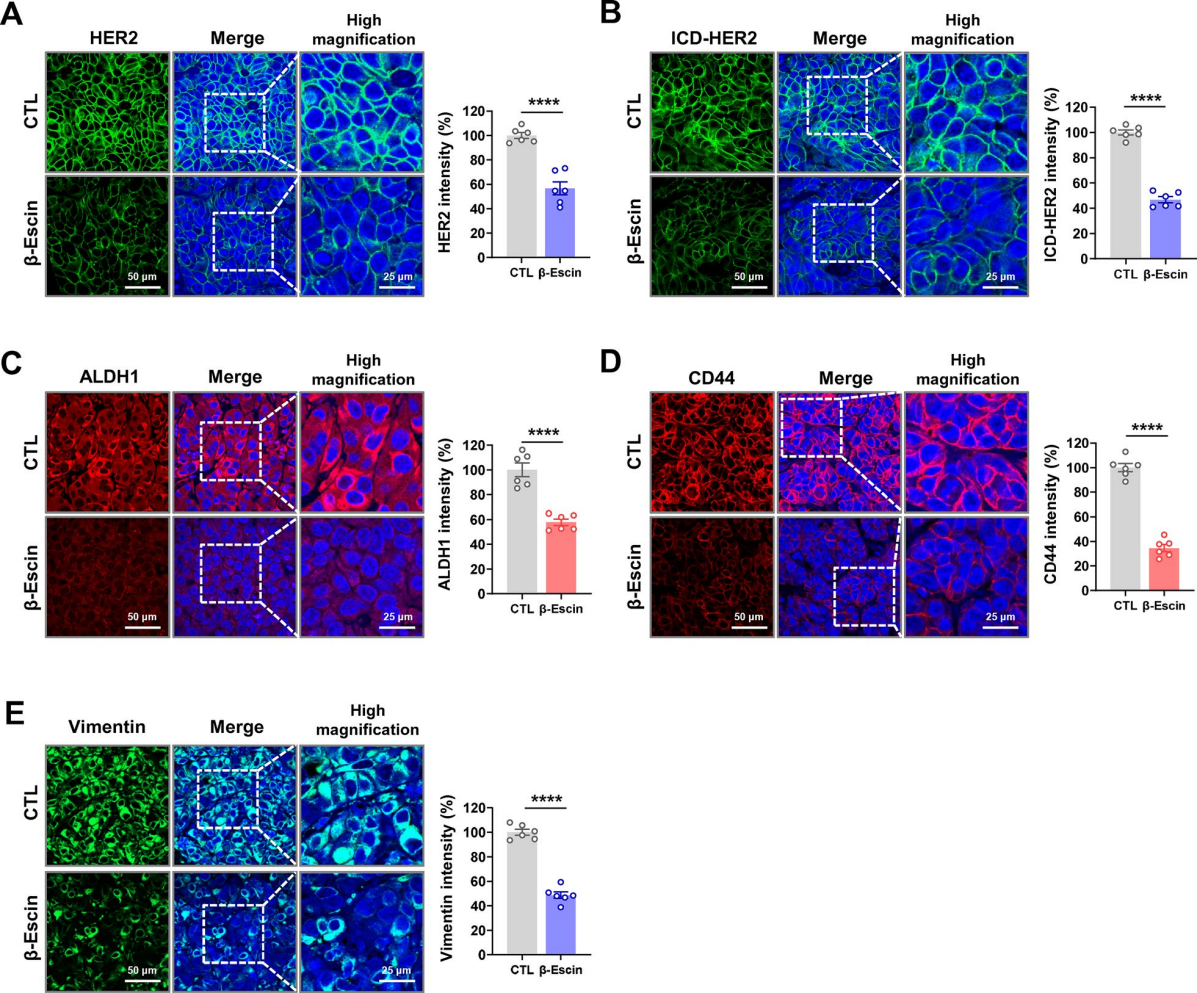
β-escin downregulates full-length HER2, ICD-HER2, ALDH1 and CD44 as well as vimentin in JIMT-1 xenograft tumors. **A**, **B** β-escin administration resulted in a marked downregulation of full-length HER2 and ICD-HER2 in JIMT-1 xenograft tumors. Tumor tissues were immunostained for full-length HER2 (polyclonal antibody 29D8, green) and ICD-HER2 (monoclonal antibody 4B5, green) with DAPI (blue). High magnification images were taken using confocal microscopy (original magnification: ×500). Quantitative graphs of signal intensities for full-length HER2 (**A**, ****p<0.0001) and ICD-HER2 (**B**, ****p<0.0001) are shown in the right panel, respectively. **C**, **D** Immunohistochemical analysis for the ALDH1 (**C**, red) and CD44 (**D**, red) in JIMT-1 xenograft tumors. Signal intensities were quantified and the graphs are shown in the right panels (****p<0.0001). **E** Influence of β-escin on vimentin expression *in vivo*. The immunofluorescence images of vimentin (green) with DAPI (blue) are shown at high magnification (×500), and the vimentin intensity was quantified (****p<0.0001). Images were taken under a confocal microscope and the fluorescence intensity was analyzed with a histogram tool within the Zen blue software. Data were analyzed using unpaired Student’s t-test

Our previous *in vitro* findings revealed that β-escin dramatically downregulates HER2 and p95HER2 levels in both trastuzumab-sensitive and -resistant HER2-positive breast cancer cells. To confirm these important observations, immunostaining analyses for full-length HER2 and intracellular domain (ICD)-HER2 were assessed in trastuzumab-resistant xenograft tumors. Expression levels of ICD-HER2 were measured using the FDA-approved antibody 4B5, which targets a specific epitope of the ICD of HER2. β-escin-treated tumors exhibited significant downregulation of HER2 (****p<0.0001, Fig. 6A and Additional file 1: Fig. S3A) and ICD-HER2 (****p<0.0001, Fig. 6B and Additional file 1: Fig. S3B) expression when compared with their control counterparts. Consistent with earlier *in vitro* observations of the eradication of BCSC-like traits, β-escin administration also elicited a considerable reduction in the expression of ALDH1 (****p<0.0001, Fig. 6C and Additional file 1: Fig. S3C) and CD44 (****p<0.0001, Fig. 6D and Additional file 1: Fig. S3D) *in vivo.*

The cytoskeletal protein vimentin is considered a major epithelial to mesenchymal transition (EMT) factor responsible for cell migration and integrity and contributes to an aggressive phenotype in breast cancer [36, 37]. Although the JIMT-1 cell line displays an epithelial-like phenotype lacking vimentin expression, we found that JIMT-1 xenograft tumors harbor vimentin-positive cells. It is conceivable that these cells gained an EMT-like phenotype during tumor development. Of particular note, vimentin was predominantly localized to the peritumoral region, comprised of cells with the highest infiltrating ability in trastuzumab-resistant JIMT-1 xenograft tumors. Immunofluorescence analysis revealed that β-escin administration caused a significant downregulation in the expression of vimentin in the peritumoral area (****p<0.0001, Fig. 6E and Additional file 1: Fig. S3E).

## Discussion

Drug repositioning is an effective strategy to apply existing drugs towards the treatment of alternate diseases. Development can benefit from existing data on the safety profile, pharmacokinetics and pharmacodynamics. Hence, the strategy can reduce the risk of process failure, shorten timelines and simplify experimental requirements and costs compared to *de novo* drugs [38]. Nevertheless, drug repurposing in cancer is an intensive endeavor due to the heterogeneous nature of cancer and the complexities of intracellular pathways. Herein, for the first time, we investigated β-escin, a drug repurposing candidate to overcome trastuzumab resistance in HER2-positive breast cancer via impairment of cancer stem-like properties and mitochondrial dysfunction. Importantly, while β-escin selectively inhibited tumor cell proliferation, it was significantly less toxic towards normal cells. Furthermore, undesirable toxic outcomes were not found in blood biochemical analysis in β-escin-treated mice, indicating that it does not affect liver or kidney health.

β-escin induced mitochondria-dependent apoptosis was accompanied by the initiation of oxidative stress and increased levels of active p18Bax, leading to the release of cytochrome c from the mitochondrial intermembrane space and consequent activation of effector caspases-3/-7. Interestingly, we found that β-escin generated a potent pro-apoptotic 18-kDa fragment. It is well known that p18Bax, a proteolytic fragment form of p21Bax arising from calpain activation, elicits more potent cytotoxicity than p21Bax [39, 40]. During apoptosis, p18Bax oligomerization is localized to the inner mitochondrial membrane, increasing its intrinsic cytotoxic properties via mitochondrial outer membrane permeabilization, release of cytochrome c and DNA fragmentation [39]. It therefore seems plausible that the increase in p18Bax by β-escin could contribute to enhanced mitochondrial dysfunction.

CSCs are capable of new tumor initiation and differentiation and are associated with a higher risk of tumor recurrence and distant metastases [41, 42]. Despite CSCs only comprising approximately 0.01-2% of the total cancer cells within a heterogeneous tumor mass, conventional anticancer treatments are rarely able to eradicate them [43]. Treatment with β-escin not only significantly eliminated the rapid proliferating tumor cells but also effectively eradicated BCSC-like populations. The latter phenomenon may be associated with the increased ROS production observed. Mitochondrial ROS are important mediators of apoptosis and augmented levels of ROS increases susceptibility to cytotoxicity [44, 45]. In particular, low ROS concentrations in CSCs appear to be important for self-renewal and maintenance, as well as for protecting their genome from oxidative damage [46, 47]. ALDH1 has antioxidant properties and contributes to CSC homeostasis, survival and protection by eliminating increased ROS after chemotherapy or radiation therapy [48, 49]. We observed that ALDH1 activity was significantly reduced in HER2-positive breast cancer cells after β-escin challenge. Therefore, it is possible that impairment of tumor stemness and ALDH1 activity by treatment with β-escin could be related to the increased ROS accumulation, and ROS-mediated anticancer strategies may lead to the clearance of CSCs.

Clinical and preclinical studies have shown that the HER2/HER3/Akt pathway is highly implicated in trastuzumab resistance, where heterodimerization of HER family members and existence of p95HER2 is associated with breast cancer treatment [6, 50]. We observed that β-escin targets HER2 and HER3, as well as the steric effects of p95HER2 and subsequently downregulates Akt in both trastuzumab-sensitive and -resistant HER2-positive breast cancer cells. High p95HER2 expression was associated with a worsening of progression-free survival (PFS) in HER2-positive metastatic breast cancer [51]. β-escin appears to downregulate p95HER2 in both trastuzumab-sensitive and -resistant cells *in vitro* as well as JIMT-1 trastuzumab-resistant xenograft tumors *in vivo*. Furthermore, we confirmed that β-escin caused a significant reduction in p95HER2-overexpressing MDA-MB-231 cells, highlighting its potential application against trastuzumab-resistant cancers. Treatment for patients expressing p95HER2 refractory to trastuzumab with the small molecule tyrosine kinase inhibitor lapatinib has been proposed as an alternative strategy to inhibit the HER2 signaling pathway [52]. Preclinical studies have shown that p95HER2-overexpressing MCF7 cells are sensitive to the tyrosine kinase inhibitor lapatinib, and lapatinib treatment resulted in suppression of cell proliferation via downregulation of p95HER2 phosphorylation and inactivation of Akt and MAPK. In the present study, both lapatinib- and trastuzumab-resistant cell lines JIMT-1 and MDA-MB-453 were used and our observations revealed that the cells were sensitive to β-escin, implying that it could be effective in overcoming cancers refractory to trastuzumab as well as lapatinib.

The inhibitory effect of β-escin on tumor growth was associated with impairment of tumoral angiogenesis *in vivo*, as evidenced by a significant decline in the number of CD31-positive microvessels in the intra- and peri-tumoral areas. Angiogenesis leads to the formation of new blood vessels and plays a pivotal role in the early stage of tumor progression [53]. Recent studies have reported that β-escin is an effective inhibitor of angiogenesis accompanied by the suppression of proliferation and migration in HUVEC cells *in vitro* as well as reducing capillary tube formation *in vivo* [54]. With its anti-angiogenic properties, β-escin may have substantial advantages in preventing cancer progression.

## Conclusions

**Fig. 7:**
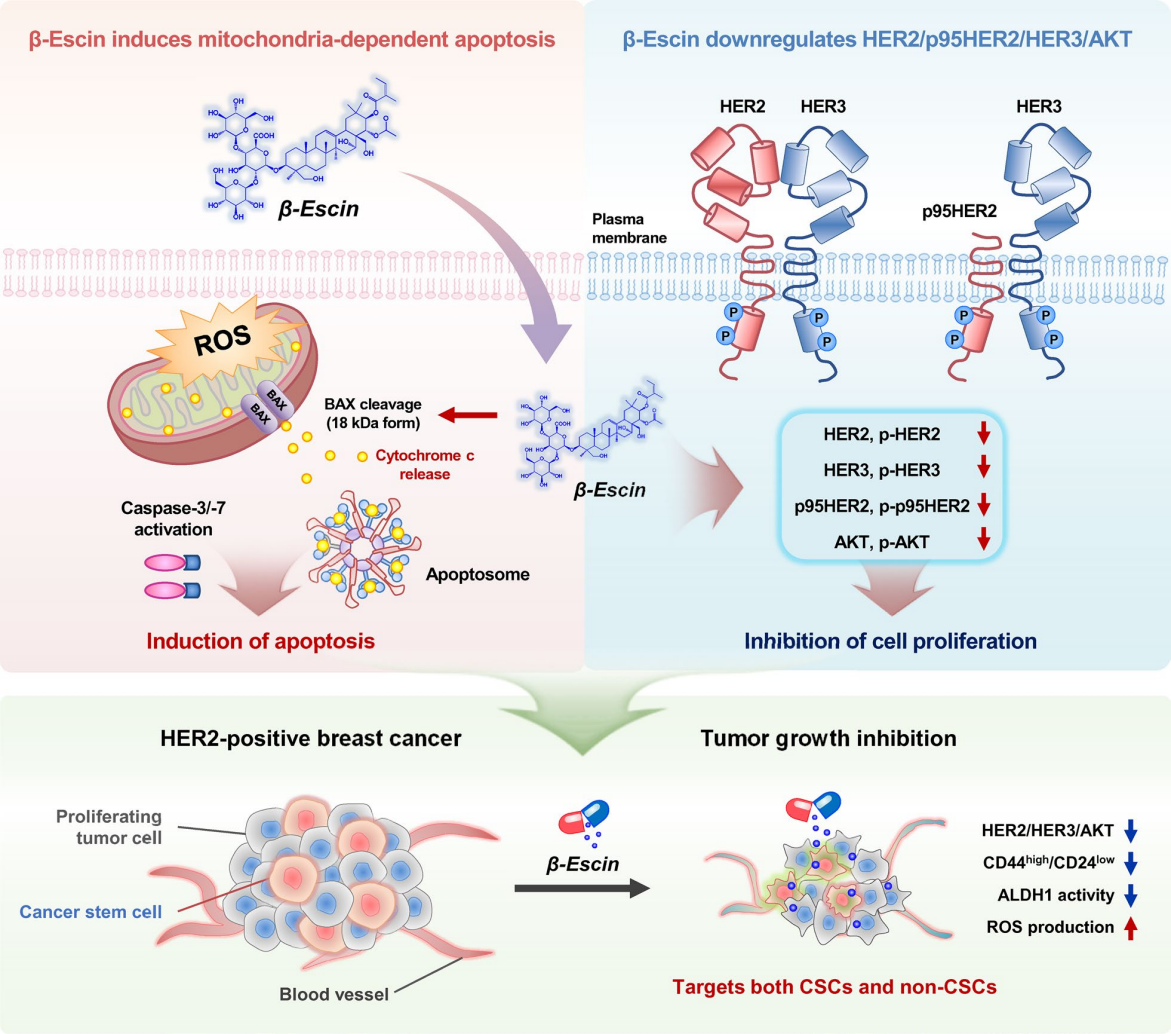
Hypothetical overview of multiple actions of β-escin on cancer stem-like features, HER2/HER3/Akt pathway and trastuzumab resistance within HER2-positive breast cancer. β-escin exerts antitumor activity via the intrinsic mitochondrial apoptotic pathway and suppression of cancer stem-like properties including CD44^high^/CD24^low^ stem-like population and ALDH1 activity. Mitochondrial-mediated apoptosis was accompanied by elevated oxidative stress and a marked increase in active p18Bax fragment levels, leading to the release of cytochrome c as well as consequent activation of caspase-3 and caspase-7. β-escin impairs the HER2/HER3/Akt pathway coinciding with downregulation of truncated-p95HER2 in HER2-positive breast cancer cells that are both trastuzumab-sensitive and –resistant

β-escin effectively targets tumor heterogeneity in HER2-positive breast cancer by deregulating mitochondrial function and attenuating tumor stemness-related factors. β-escin effectively suppresses tumor growth and angiogenesis, with no evidence of toxicity found in terms of liver and kidney function. Due to inactivation of the HER2/HER3/Akt pathway and anti-CSC properties, further investigation of β-escin is warranted to understand its role in addressing trastuzumab resistance in HER2-positive breast cancers (Fig. 7).

## Supplementary Information

**Additional file 1:** **Figure. S1.** Effect of β-escin on HER2 and p95HER2 expression, corresponding to Fig. 3E, F, H and I in the main text. (**A**, **B**) Immunofluorescence analysis of HER2 (red) or normal rabbit IgG (as a negative control) with DAPI (nuclei, blue) in BT474 (**A**) and JIMT-1 cells (**B**) after treatment with β-escin (20 and 30 μM, respectively) for 24 h. Intensity profiles represent HER2 expression with green signal fluorescence. (**C**, **D**) Immunofluorescence analyses of HER2 (**C**, green) and ICD-HER2 (**D**, green, 4B5) or normal rabbit IgG (as a negative control) with DAPI (nucleus, blue) in HER2- and p95HER2-overexpressing MDA-MB-231 cells in the presence of β-escin (20 and 30 μM, respectively) for 24 h. Intensity profiles were analyzed with the histogram tool in the Zen blue software and the horizontal line (white dotted line) indicates 100 intensity units (range of y-axis, 0-250 units). **Figure. S2.** Influence of β-escin on Ki-67 expression, apoptosis and tumor angiogenesis in vivo, corresponding to Fig. 5I-K in the main text. (**A**) The effect of β-escin on Ki-67 expression. Sections were immunostained to assess Ki-67 (red) or normal rabbit IgG (as a negative control) with DAPI (blue). Original magnification: × 500. (**B**) β-escin-induced apoptosis was determined by TUNEL assay. The white arrows indicate the TUNEL-positive apoptotic cells. Label solution without terminal transferase enzyme was used as a negative control. Original magnification: × 500. (**C**) Tumor angiogenesis was evaluated for each group of xenograft tumors. Tissues were immunostained using an endothelial cell marker CD31 (red) or normal rabbit IgG (as a negative control) with DAPI (blue). Original magnification: × 250. **Figure. S3.** Effect of β-escin on full-length HER2, ICD-HER2, ALDH1, CD44 and vimentin in JIMT-1 xenograft tumors, corresponding to Fig. 6A-E in the main text. (**A**, **B**) β-escin administration resulted in a marked downregulation of full-length HER2 and ICD-HER2 in JIMT-1 xenograft tumors. Tumor tissues were immunostained for full-length HER2 (polyclonal antibody 29D8, green), ICD-HER2 (monoclonal antibody 4B5, green) or normal rabbit IgG (as a negative control) with DAPI (blue). High magnification images were taken using confocal microscopy (original magnification: ×500). Quantitative graphs of signal intensities for full-length HER2 (A, ****p<0.0001) and ICD-HER2 (**B**, ****p<0.0001) are shown in the right panel, respectively. (**C**, **D**) Immunohistochemical analysis for the ALDH1 (**C**, red), CD44 (**D**, red) or normal rabbit IgG (as a negative control) in JIMT-1 xenograft tumors. Signal intensities were quantified and the graphs are shown in the right panels (****p<0.0001). (**E**) Influence of β-escin on vimentin expression in vivo. The immunofluorescence images of vimentin (green) or normal rabbit IgG (as a negative control) with DAPI (blue) are shown at high magnification (×500), and the vimentin intensity was quantified (****p<0.0001). Images were taken under a confocal microscope and the fluorescence intensity was analyzed with a histogram tool within the Zen blue software. Data were analyzed using unpaired Student’s ttest.

## Data Availability

Not applicable.

## Abbreviations

ALDH1: Aldehyde dehydrogenase 1
ALT: Alanine aminotransferase
AST: Aspartate aminotransferase
BCSC: Breast cancer stem cell
bFGF: Basic fibroblast growth factor
BUN: Blood urea nitrogen
CD31: Cluster of differentiation 31
CD44: Cluster of differentiation 44
CSC: Cancer stem cell
CTL: Control
DAPI: 4′,6-Diamidino-2-phenylindole
DCF: 2′, 7′- Dichlorodihydrofluorescein
DCFH-DA: 2′, 7′-Dichlorodihydrofluorescin diacetate
DEAB: Diethylamino-benzaldehyde
DMSO: Dimethyl sulfoxide
EGFR: Epidermal growth factor receptor
GAPDH: Glyceraldehyde 3-phosphate dehydrogenase
hEGF: Human epidermal growth factor
HER2: Human epidermal growth factor receptor 2
ICD: Intracellular domain
H&E: Hematoxylin and eosin
HRP: Horseradish peroxidase
MAPK: A mitogen-activated protein kinase
MTS: 3-(4,5-Dimethylthiazol-2-yl)-5-(3-carboxymethoxyphenyl)-2-(4-sulfophenyl)-2H-tetrazolium
NANOG: Nanog homeobox
OCT4: Octamer-binding transcription factor 4
PARP: Poly (ADP-ribose) polymerase
PI: Propidium iodide
ROS: Reactive oxygen species
SOX2: SRY-Box transcription factor 2
TNBC: Triple-negative breast cancer
TOM 20: Translocase of outer membrane 20
