# Supplementary material for: β-Escin overcomes trastuzumab resistance in HER2-positive breast cancer by targeting cancer stem-like features
Source: Cancer Cell Int. 2022 Sep 20;22:289. doi: 10.1186/s12935-022-02713-9 (PMC9490928; doi:10.1186/s12935-022-02713-9)
Supplement: Supplementary file 1 — Additional file 1: Figure. S1. Effect of β-escin on HER2 and p95HER2 expression, corresponding to Fig. 3E, F, H and I in the main text. (A, B) Immunofluorescence analysis of HER2 (red) or normal rabbit IgG (as a negative control) with DAPI (nuclei, blue) in BT474 (A) and JIMT-1 cells (B) after treatment with β-escin (20 and 30 μM, respectively) for 24 h. Intensity profiles represent HER2 expression with green signal fluorescence. (C, D) Immunofluorescence analyses of HER2 (C, green) and ICD-HER2 (D, green, 4B5) or normal rabbit IgG (as a negative control) with DAPI (nucleus, blue) in HER2- and p95HER2-overexpressing MDA-MB-231 cells in the presence of β-escin (20 and 30 μM, respectively) for 24 h. Intensity profiles were analyzed with the histogram tool in the Zen blue software and the horizontal line (white dotted line) indicates 100 intensity units (range of y-axis, 0-250 units). Figure. S2. Influence of β-escin on Ki-67 expression, apoptosis and tumor angiogenesis in vivo, corresponding to Fig. 5I-K in the main text. (A) The effect of β-escin on Ki-67 expression. Sections were immunostained to assess Ki-67 (red) or normal rabbit IgG (as a negative control) with DAPI (blue). Original magnification: × 500. (B) β-escin-induced apoptosis was determined by TUNEL assay. The white arrows indicate the TUNEL-positive apoptotic cells. Label solution without terminal transferase enzyme was used as a negative control. Original magnification: × 500. (C) Tumor angiogenesis was evaluated for each group of xenograft tumors. Tissues were immunostained using an endothelial cell marker CD31 (red) or normal rabbit IgG (as a negative control) with DAPI (blue). Original magnification: × 250. Figure. S3. Effect of β-escin on full-length HER2, ICD-HER2, ALDH1, CD44 and vimentin in JIMT-1 xenograft tumors, corresponding to Fig. 6A-E in the main text. (A, B) β-escin administration resulted in a marked downregulation of full-length HER2 and ICD-HER2 in JIMT-1 xenograft tumors. Tumor tissue [file 12935_2022_2713_MOESM1_ESM.pdf]

Supplementary information

Title: β-Escin overcomes trastuzumab resistance in HER2-positive breast cancer by targeting cancer stem-like features

Corresponding authors: Yoon-Jae Kim, Ji Young Kim and Jae Hong Seo

Supplementary Figures and Legends (Fig. S1-S3)

Supplementary Fig. S1

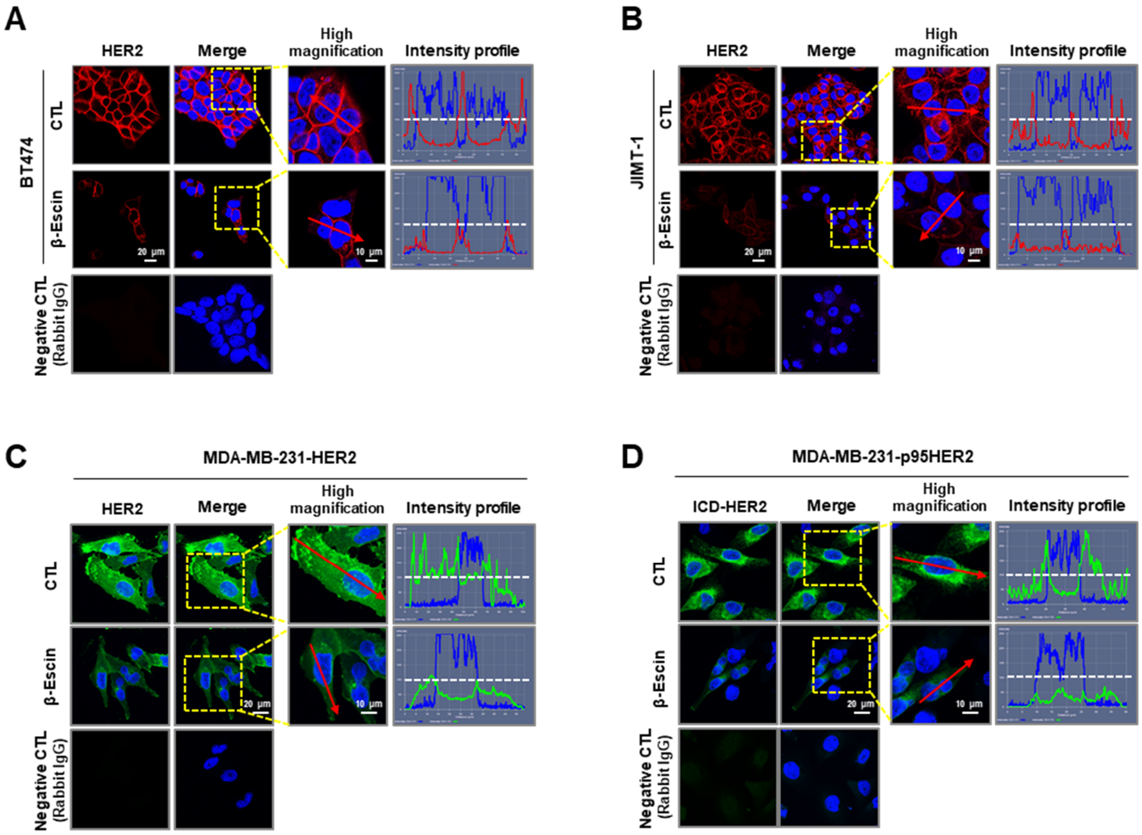

Supplementary Fig. S1. Effect of β-escin on HER2 and p95HER2 expression, corresponding to Fig. 3E, F, H and I in the main text. (**A, B**) Immunofluorescence analysis of HER2 (red) or normal rabbit IgG (as a negative control) with DAPI (nuclei, blue) in BT474 (**A**) and JIMT-1 cells (**B**) after treatment with β-escin (20 and 30 μM, respectively) for 24 h. Intensity profiles represent HER2 expression with green signal fluorescence. **(C, D)** Immunofluorescence analyses of HER2 (**C**, green) and ICD-HER2 (**D**, green, 4B5) or normal

rabbit IgG (as a negative control) with DAPI (nucleus, blue) in HER2- and p95HER2-overexpressing MDA-MB-231 cells in the presence of β-escin (20 and 30 μM, respectively) for 24 h. Intensity profiles were analyzed with the histogram tool in the Zen blue software and the horizontal line (white dotted line) indicates 100 intensity units (range of y-axis, 0-250 units).

Supplementary Fig. S2

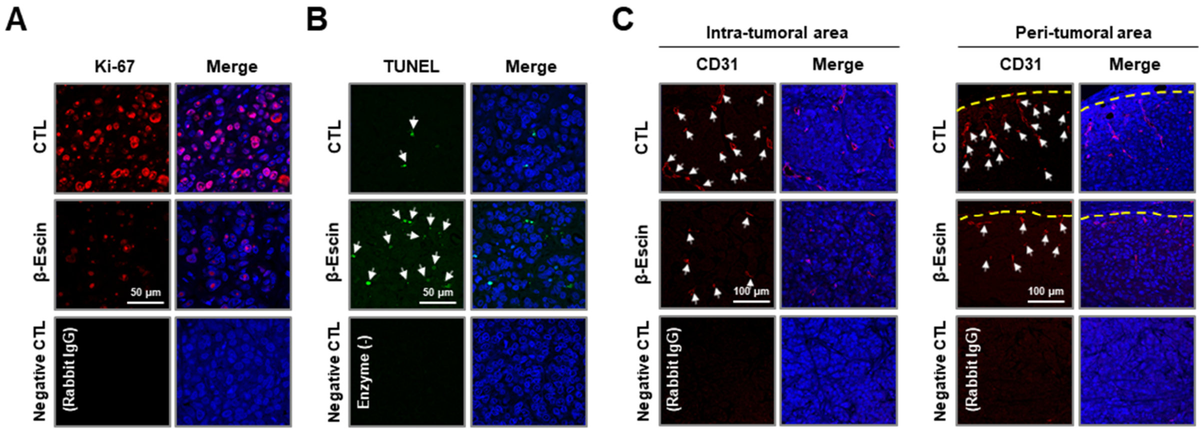

Supplementary Fig. S2. Influence of β-escin on Ki-67 expression, apoptosis and tumor angiogenesis *in vivo*, corresponding to Fig. 5I-K in the main text. (**A**) The effect of β-escin on Ki-67 expression. Sections were immunostained to assess Ki-67 (red) or normal rabbit IgG (as a negative control) with DAPI (blue). Original magnification: × 500. (**B**) β-escin-induced apoptosis was determined by TUNEL assay. The white arrows indicate the TUNEL-positive apoptotic cells. Label solution without terminal transferase enzyme was used as a negative control. Original magnification: × 500. (**C**) Tumor angiogenesis was evaluated for each group of xenograft tumors. Tissues were immunostained using an endothelial cell marker CD31 (red) or normal rabbit IgG (as a negative control) with DAPI (blue). Original magnification: × 250.

Supplementary Fig. S3

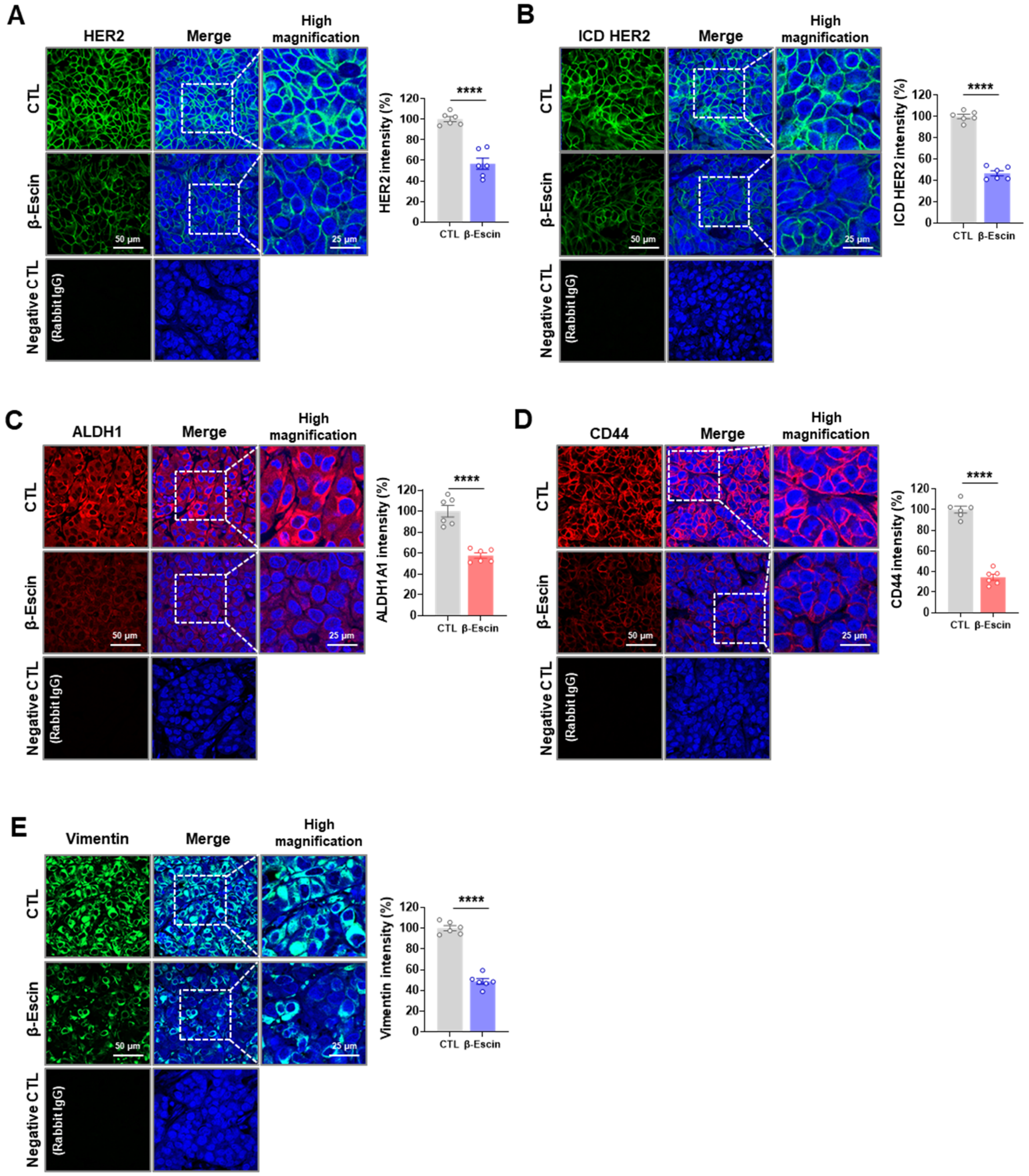

Supplementary Fig. S3. Effect of β-escin on full-length HER2, ICD-HER2, ALDH1, CD44 and vimentin in JIMT-1 xenograft tumors, corresponding to Fig. 6A-E in the main text. (**A, B**) β-escin administration resulted in a marked downregulation of full-length HER2 and ICD-HER2 in JIMT-1 xenograft tumors. Tumor tissues were immunostained for full-length HER2 (polyclonal antibody 29D8, green), ICD-HER2 (monoclonal antibody 4B5, green) or

normal rabbit IgG (as a negative control) with DAPI (blue). High magnification images were taken using confocal microscopy (original magnification: ×500). Quantitative graphs of signal intensities for full-length HER2 (**A**, ****$p<0.0001$) and ICD-HER2 (**B**, ****$p<0.0001$) are shown in the right panel, respectively. (**C, D**) Immunohistochemical analysis for the ALDH1 (**C**, red), CD44 (**D**, red) or normal rabbit IgG (as a negative control) in JIMT-1 xenograft tumors. Signal intensities were quantified and the graphs are shown in the right panels (****$p<0.0001$). (**E**) Influence of β-escin on vimentin expression *in vivo*. The immunofluorescence images of vimentin (green) or normal rabbit IgG (as a negative control) with DAPI (blue) are shown at high magnification (×500), and the vimentin intensity was quantified (****$p<0.0001$). Images were taken under a confocal microscope and the fluorescence intensity was analyzed with a histogram tool within the Zen blue software. Data were analyzed using unpaired Student's t-test.
